# PET Imaging with *S*-[^11^C]Methyl-L-Cysteine and L-[*Methyl*-^11^C]Methionine in Rat Models of Glioma, Glioma Radiotherapy, and Neuroinflammation

**DOI:** 10.1007/s11307-017-1137-z

**Published:** 2017-10-30

**Authors:** Andrea Parente, Aren van Waarde, Alexandre Shoji, Daniele de Paula Faria, Bram Maas, Rolf Zijlma, Rudi A. J. O. Dierckx, Johannes A. Langendijk, Erik F.J. de Vries, Janine Doorduin

**Affiliations:** 1 Nuclear Medicine and Molecular Imaging, University of Groningen, University Medical Center Groningen, Hanzeplein 1, 9713 GZ Groningen, The Netherlands; 2 Laboratory of Nuclear Medicine (LIM43), University of Sao Paulo, Faculdade de Medicina, Hospital das Clinicas, Sao Paulo, SP Brazil; 3 Department of Radiation Oncology, University of Groningen, University Medical Center Groningen, Hanzeplein 1, 9713 GZ Groningen, The Netherlands

**Keywords:** Amino acids, Brain tumors, Inflammation, Brain, Positron emission tomography, Small animal imaging

## Abstract

**Purpose:**

S-[^11^C]-methyl-L-cysteine ([^11^C]MCYS) has been claimed to offer higher tumor selectivity than L-[*methyl-* ^11^C]methionine ([^11^C]MET). We examined this claim in animal models.

**Procedures:**

Rats with implanted untreated (*n* = 10) or irradiated (*n* = 7, 1 × 25 Gy, on day 8) orthotopic gliomas were scanned after 6, 9, and 12 days, using positron emission tomography. Rats with striatal injections of saline (*n* = 9) or bacterial lipopolysaccharide (*n* = 9) were scanned after 3 days.

**Results:**

Uptake of the two tracers in untreated gliomas was similar. [^11^C]MCYS was not accumulated in salivary glands, nasal epithelium, and healing wounds, in contrast to [^11^C]MET, but showed 40 % higher accumulation in the healthy brain. Both tracers showed a reduced tumor uptake 4 days after irradiation and minor accumulation in inflamed striatum. [^11^C]MCYS indicated higher lesion volumes than [^11^C]MET (untreated tumor + 47 %; irradiated tumor up to + 500 %; LPS-inflamed striatum + 240 %).

**Conclusions:**

[^11^C]MCYS was less accumulated in some non-tumor tissues than [^11^C]MET, but showed lower tumor-to-brain contrast.

**Electronic supplementary material:**

The online version of this article (10.1007/s11307-017-1137-z) contains supplementary material, which is available to authorized users.

## Introduction

The radiolabeled amino acid L-[*methyl*-^11^C]methionine ([^11^C]MET) is widely used for tumor imaging. However, [^11^C]MET is taken up not only by tumors but also by various other lesions [1–3]. For this reason, there is much interest in the development of positron emission tomography (PET) tracers with greater tumor specificity than [^11^C]MET. A successful alternative is the amino acid analog *O*-2-[^18^F]fluoroethyl-L-tyrosine ([^18^F]FET) [4]. Another amino acid analog which has been proposed for the same purpose is *S*-[^11^C]methyl-L-cysteine ([^11^C]MCYS) [5, 6]. MCYS is a naturally occurring derivative of the amino acid L-cysteine. Large amounts of this substance are present in many vegetables [7, 8]. Biodistribution studies in nude mice indicated a somewhat higher uptake of [^11^C]MCYS in Hepa1–6 tumors compared to [^11^C]MET, but a lower uptake in turpentine-induced sterile inflammations [5]. Thus, [^11^C]MCYS was claimed to offer superior differentiation of tumor from inflammation and to have considerable potential as an oncologic PET tracer.

In order to examine this claim, we performed longitudinal PET studies with [^11^C]MCYS and [^11^C]MET in various animal models: immune-competent rats with orthotopically implanted gliomas (see [9]) which were either untreated or received radiotherapy, rats which were injected with bacterial lipopolysaccharide (LPS) in the right striatum (see [10]), and sham-injected rats which received physiological saline instead of LPS. Our data indicate that [^11^C]MCYS accumulates less in some non-tumor tissues (salivary glands, Harderian glands, nasal epithelium, healing wounds) than [^11^C]MET, but shows a higher uptake in the healthy brain, resulting in lower tumor-to-brain contrast of [^11^C]MCYS scans. Moreover, irradiation of gliomas results in an acute, 5- to 6-fold increase of the volume with significant [^11^C]MCYS uptake which does not reflect the presence of viable tumor cells. Based on these results, [^11^C]MCYS and [^11^C]MET appear to reflect different aspects of *in vivo* biology.

## Materials and Methods

### Reagents

L-[*methyl*-^11^C]methionine was prepared by methylation of L-homocysteine thiolactone with [^11^C]methyl iodide. The radiochemical yield was 60 %, and specific activities were greater than 2 TBq/mmol [1]. S-[^11^C]methyl-L-cysteine was synthesized by [^11^C]methylation of L-cysteine [5], using [^11^C]methyl triflate rather than [^11^C]methyl iodide. 2-Deoxy-2-[^18^F]fluoro-D-glucose ([^18^F]FDG) was produced by the Hamacher method [11]. Bacterial lipopolysaccharide (LPS) was purchased from Sigma Aldrich (cat.no. L6529).

### Animals

All actions with experimental animals were performed by licensed investigators. The study protocol was approved by the Institutional Animal Care and Use Committee of the University of Groningen (protocol 6561A). Male Wistar rats were acquired from Harlan. They were housed at 21 ± 2 °C under a fixed 12-h light–dark regime. Standard laboratory chow and water were available *ad libitum*. The animals received a special high-energy diet on the day of the surgery and the following day, as well as 2 days before and after brain irradiation (see below). After a 7-day acclimation period, the animals were randomly divided in five different groups (see Table 1): *1* (*Pilot*): C6 glioma cells were stereotactically injected in the right striatum. [^11^C]MCYS and [^11^C]MET scans were made after 8 days and a [^18^F]FDG scan after 15 days; *2* (*Tumor-bearing*, *untreated*): C6 glioma cells were injected as in group 1, but [^11^C]MCYS and [^11^C]MET scans were made after 6, 9, and 12 days; *3* (*Tumor-bearing*, *radiotherapy*): As group 2, but the lesion-containing hemisphere was irradiated after 8 days; *4* (*Saline-injected*): 2 μl of physiological saline was stereotactically injected into the right striatum and [^11^C]MCYS and [^11^C]MET scans were made after 3 days; *5* (*LPS-injected*): As group 4, but 2 μl of a solution of LPS in saline was stereotactically injected into the right striatum in order to induce neuroinflammation.

**Table 1:** Groups included in this study

Group	Number of rats	Intervals between inoculation/injection and PET scan	Interval between inoculation and radiotherapy	X-ray dose (Gy)	Body weight (g)	[^11^C]MET (MBq)	[^11^C]MCYS (MBq)
1. Tumor-bearing (pilot study)	4	8 and 15 days	–	0	278 ± 8	27 ± 17	21 ± 13
2. Tumor-bearing (untreated)	10	6, 9 and 12 days	–	0	314 ± 23	23 ± 12	24 ± 13
3. Tumor-bearing (radiotherapy)	7	6, 9 and 12 days	8 days	25	308 ± 13	21 ± 6	22 ± 5
4. Right striatum injected with saline	9	3 days	–	0	283 ± 19	33 ± 20	31 ± 17
5. Right striatum injected with LPS	9	3 days	–	0	283 ± 17	34 ± 19	28 ± 17

On all mentioned days, [^11^C]MET and [^11^C]MCYS scans were made in each rat, with exception of day 15 (group 1) when [^18^F]FDG was used

### Stereotactic Injection of C6 Cells

Rats from groups 1, 2, and 3 were anesthetized by intraperitoneal injection of ketamine (25 mg/kg) and medetomidine (0.2 mg/kg) and placed in a stereotactic frame. Eye cream was applied and heating pads were used to maintain body temperatures close to the normal value. During surgery, the rats breathed oxygen from a nose mask, and pO_2_ and heart rate were continuously monitored. A longitudinal incision was made along the medial line of the skull, the skin and fascia were pushed aside, and the skull was exposed. A hole was drilled at the coordinates of the striatum (from Bregma *A* = − 0.30; *L* = + 3.0), until the dura became visible and could be opened [12]. Using a Hamilton injection needle, 5 × 10^5^ C6 cells in 5 μl of sterile saline were slowly injected (in 10 min), at a depth of − 5.0 from Bregma. Before injection, the needle was wiped with 70 % alcohol and saline to avoid contamination of host tissue with tumor cells outside the desired area. The syringe was left in position for an additional 5 min and was then withdrawn. The skin was closed and saline was injected subcutaneously to prevent dehydration. Bupivacaine (5 mg/ml) was locally applied to suppress pain. Anti-sedan was used to wake up the animal and 0.03 mg/kg of s.c. Temgesic was given after 30 min.

### Irradiation

Group 3 received special care to reduce the discomfort of radiotherapy as much as possible, starting 2 days before irradiation. This special treatment involved the feeding of a high-energy diet, the addition of sucrose to the drinking water, and the application of Bepanthen® cream to the skull after radiotherapy. On the day of treatment, rats were anesthetized by intraperitoneal injection of ketamine (25 mg/kg) and xylazine (20 mg/ml). Eye cream was applied, and the animals were placed in a special frame containing a lead collimator which shielded the non-lesioned hemisphere and all tissues outside the brain, including the eyes and the parotid glands. The right hemisphere was irradiated with a single X-ray fraction of 25 Gy. After irradiation (about 18 min), the rats were aroused with Anti-sedan, returned to their cages, and allowed to recover. The entire procedure was finished within 30 min.

### Stereotactic Injection of Saline or LPS

Rats were injected with saline or LPS using the same procedure as for injection of C6 cells, but the injected volume was 2 μl and the sterile saline contained either nothing (group 4) or bacterial lipopolysaccharide (*Escherichia coli* 020:B6, 0.5 μg/μl, group 5).

### PET Imaging

On each scanning day, rats were anesthetized with isoflurane (2 % in medical air, flow rate 1 to 2 ml/min). [^11^C]MCYS or [^11^C]MET was injected *via* a tail or penile vein. A dynamic emission scan of 60 min was made with a Siemens/Concorde Focus 220 camera, using a list mode protocol. Two rats were scanned simultaneously, in transaxial position with their brains in the field-of-view. In some animals, a static rather than a dynamic scan was made for logistic reasons, lasting from 30 to 60 min after tracer injection. Finally, a transmission scan was made (515 s), using a Co-57 point source. Data from this scan were used for the correction of attenuation and scatter of 511-keV photons by tissue. During all scans, body temperature of the animals was maintained by heating mats, whereas heart rate and blood oxygenation were continuously monitored. A [^11^C]MCYS scan and a [^11^C]MET scan were made of each animal on each scanning day, with an interval of at least 2 h. The list mode data of the emission scans were reframed into a dynamic sequence: 6 × 10 s, 4 × 30 s, 2 × 60 s, 1 × 120 s, 1 × 180 s, 4 × 300 s, 3 × 600 s frames. Images were reconstructed employing ordered subset expectation maximization (OSEM 2D with Fourier rebinning, four iterations, and 16 subsets). The final datasets consisted of 95 slices with a thickness of 0.8 mm and an in-plane image matrix of 128 × 128 pixels. Voxel size was 0.5 × 0.5 × 0.8 mm. The linear resolution at the center of the field-of-view was 1.5 mm. Data sets were corrected for decay, random coincidences, scatter, and attenuation.

### Data Analysis

Three-dimensional regions of interest (ROIs) were manually drawn in PET images, representing the tumor, LPS- or saline-injected striatum, contralateral healthy brain, and the wound on top of the skull. ROI volumes and levels of radioactivity (mean and maximum within the ROI) were calculated, using AsiPro® (Siemens). Tracer accumulation was expressed as a standardized uptake value (SUV): [tissue activity concentration (MBq/g) × body weight (g)/injected dose (MBq)], assuming a specific tissue gravity of 1 g/ml. Images were smoothed with a Gaussian filter (1.5 mm in both directions). The color scale of the images was set from SUV 0 to 2 (± 10 %), in order to clearly delineate the lesion. However, for visualization of LPS- or saline-injected striatum with [^11^C]MET, the maximum had to be set to SUV 1.4 (± 10 %). Care was taken to include in the ROIs only planes that were located within the brain, since wound tissue on top of the skull could strongly take up [^11^C]MET, particularly at short intervals (3–6 days) after surgery.

### Statistics

Results reported in the Tables are expressed as mean ± SD. Error bars represent SEM. Group differences were examined using *t* test and two-way ANOVA, followed by a Bonferroni correction, where applicable. A *P* value < 0.05 was considered statistically significant.

## Results

### Group 1

The pilot study confirmed that inoculation of C6 cells led to the formation of a tumor that was clearly visible after 8 days (volume ~ 60 μl). An [^18^F]FDG scan made in the same rats after 15 days indicated the presence of tumors with a volume of 451 ± 81 μl, filling almost an entire cerebral hemisphere and starting to invade the rest of the brain (Figure in Supplementary Evidence). Because of this rapid growth, we decided to terminate our animals after 12 rather than 15 days. An interval of 8 days was selected for the application of radiotherapy whereas [^11^C]MET and [^11^C]MCYS scans in groups 2 and 3 were made at 6, 9, and 12 days.

### Group 2

[^11^C]MCYS and [^11^C]MET showed a similar uptake in untreated tumors at 6, 8, or 9 days post inoculation (Table 2). At 12 days, [^11^C]MCYS showed a 14 to 20 % higher uptake than [^11^C]MET (Table 2). The uptake of [^11^C]MCYS in healthy brain tissue was always 40 % higher than that of [^11^C]MET (*P* < 0.0001, Table 2). As a consequence of this higher brain uptake, tumor-to-brain ratios of [^11^C]MCYS were 20 to 30 % lower than those of [^11^C]MET (*P* < 0.0001, Table 2).

**Table 2:** Tracer uptake in rats with untreated brain tumors

Tracer uptake (SUV)		Tumor				*N*	Brain	
Time	*N*	[^11^C]MET SUVmean	[^11^C]MCYS SUVmean	[^11^C]MET SUVmax	[^11^C]MCYS SUVmax		[^11^C]MET SUVmean	[^11^C]MCYS SUVmean
6 days p.i.	10	0.96 ± 0.17	1.00 ± 0.09	1.22 ± 0.29	1.24 ± 0.28	34	0.59 ± 0.07	0.83 ± 0.07*
8 days p.i.	4	1.24 ± 0.09	1.19 ± 0.03	1.76 ± 0.10	1.75 ± 0.20			
9 days p.i.	10	1.26 ± 0.13	1.24 ± 0.13	1.92 ± 0.57	1.79 ± 0.40			
12 days p.i.	10	1.18 ± 0.10	1.35 ± 0.13^@^	1.77 ± 0.51	2.13 ± 0.43^$^			
Uptake ratios		Tumor-to-brain				*N*	Wound-to-brain	
	*N*	[^11^C]MET Mean ratio	[^11^C]MCYS Mean ratio	[^11^C]MET Max ratio	[^11^C]MCYS Max ratio		[^11^C]MET Mean ratio	[^11^C]MCYS Mean ratio
6 days p.i.	10	1.72 ± 0.24	1.31 ± 0.10*	1.96 ± 0.32	1.53 ± 0.35^@^	10	1.61 ± 0.16	0.95 ± 0.13*
8 days p.i.	4	1.99 ± 0.16	1.40 ± 0.09^#^	2.46 ± 0.36	1.91 ± 0.27^$^			
9 days p.i.	10	2.05 ± 0.20	1.45 ± 0.12*	2.61 ± 0.57	1.92 ± 0.39^@^			
12 days p.i.	10	2.07 ± 0.28	1.59 ± 0.11*	2.59 ± 0.74	2.28 ± 0.46			
Overall	34	1.95 ± 0.27	1.45 ± 0.15*	2.40 ± 0.60	1.91 ± 0.47*			

Data for 8 days p.i. are from a separate pilot study. “Wound” refers to tissue on top of the skull. “Mean ratio” indicates SUVmean in tumor/SUVmean in brain or SUVmean in wound/SUVmean in brain. “Max ratio” indicates SUVmax in tumor/SUVmax in brain

*P.i.* post inoculation

*P < 0.0001, ^#^ *P* < 0.001, ^@^ *P* < 0.005, and ^$^ *P* < 0.05 indicate significant differences between [^11^C]MET and [^11^C]MCYS

**Fig. 1:**
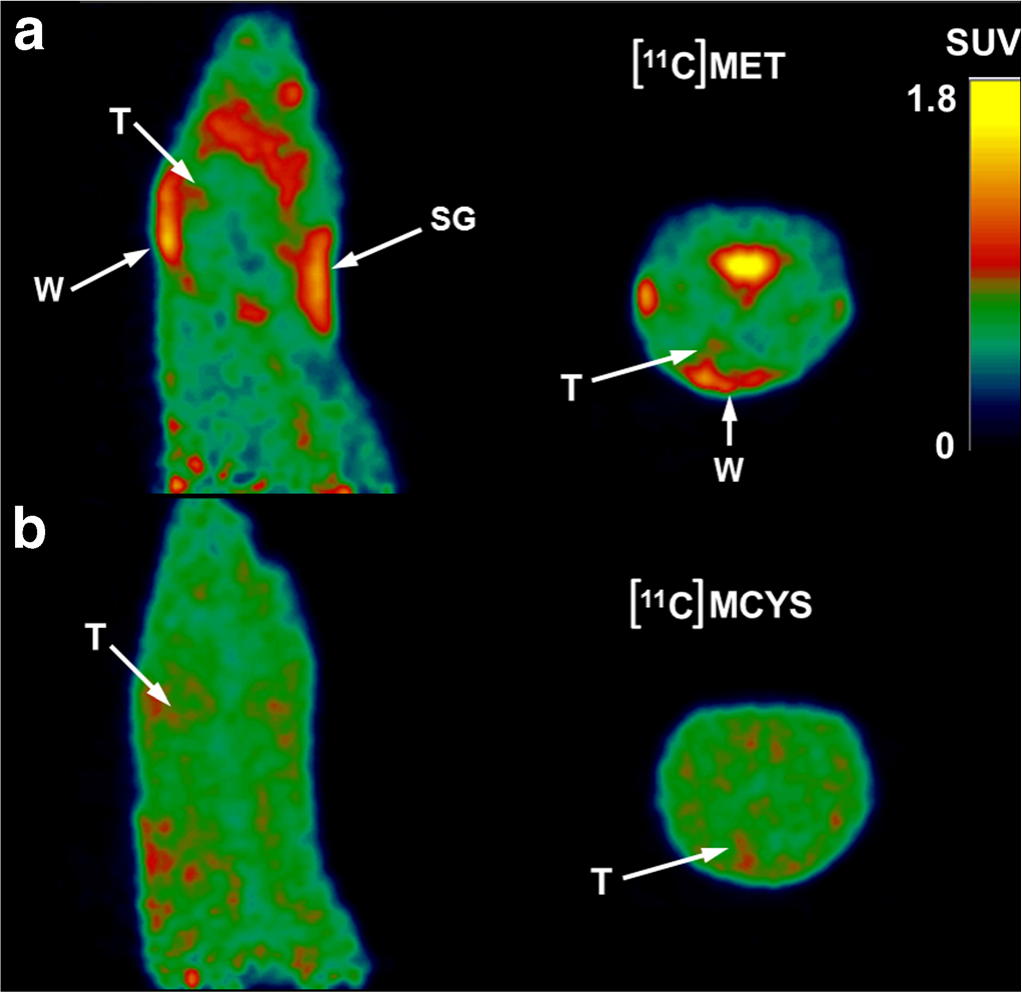
PET images of a single rat made 6 days after inoculation of tumor cells. (**a**) [^11^C]MET is strongly accumulated in the healing wound (W) on top of the skull and also in submandibular gland (SG) in contrast to (**b**) [^11^C]MCYS. The tumor (T) is still very small and barely visible.

[^11^C]MET was strongly accumulated in parotid and submandibular glands, nasal epithelium, Harderian glands, and healing wounds (including the injection spot in the skull), but [^11^C]MCYS showed only minor accumulation in these tissues (Fig. 1). At 6 days after surgery, [^11^C]MET was significantly accumulated in the wound, in contrast to [^11^C]MCYS (wound-to-brain ratio 1.61 ± 0.16 *vs* 0.95 ± 0.13, Table 2). This difference between the tracers became smaller at later time points. Apparently, the surgical trauma was gradually healed.

**Fig. 2:**
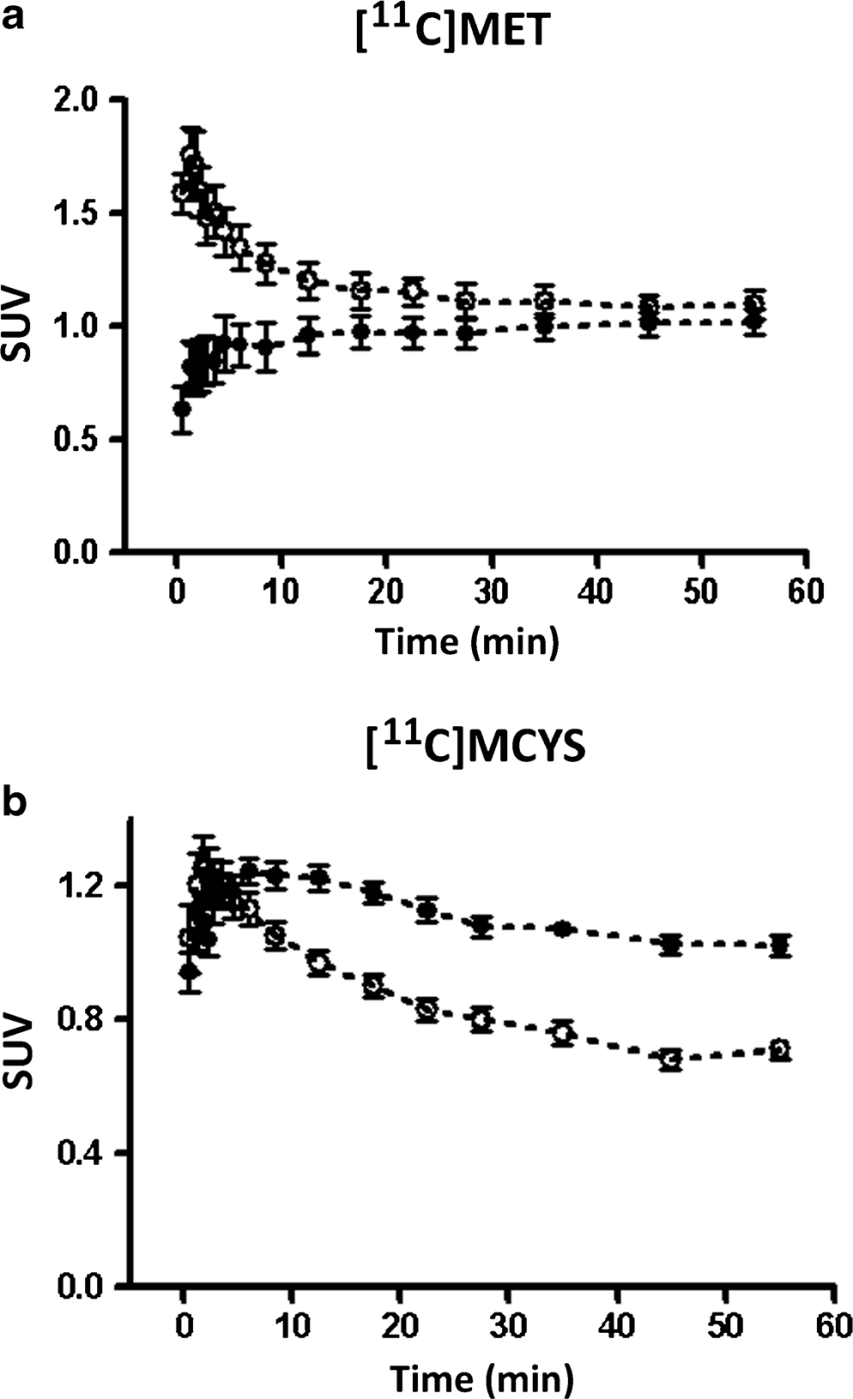
Kinetics of (**a**) [^11^C]MET and (**b**) [^11^C]MCYS derived radioactivity in tumor (●) and in wound tissue (○).

Tumor and wound tissue showed different tracer kinetics (Fig. 2). After injection of [^11^C]MET, radioactivity in the tumor slowly increased and reached a plateau between 30 and 60 min. In the wound on top of the skull, radioactivity peaked within 1 min and subsequently showed a partial washout. During the static scans (30–60 min), uptake of radioactivity in tumor and wound tissue was about equal (Fig. 2a). After injection of [^11^C]MCYS, radioactivity in the tumor reached a maximum between 3 and 15 min, whereas a minor washout was observed between 15 and 60 min. Radioactivity in the wound peaked at 2 min and subsequently showed a partial washout. During the static scans, uptake of radioactivity in the tumor was significantly greater than in the healing wound (Fig. 2b).

**Fig. 3:**
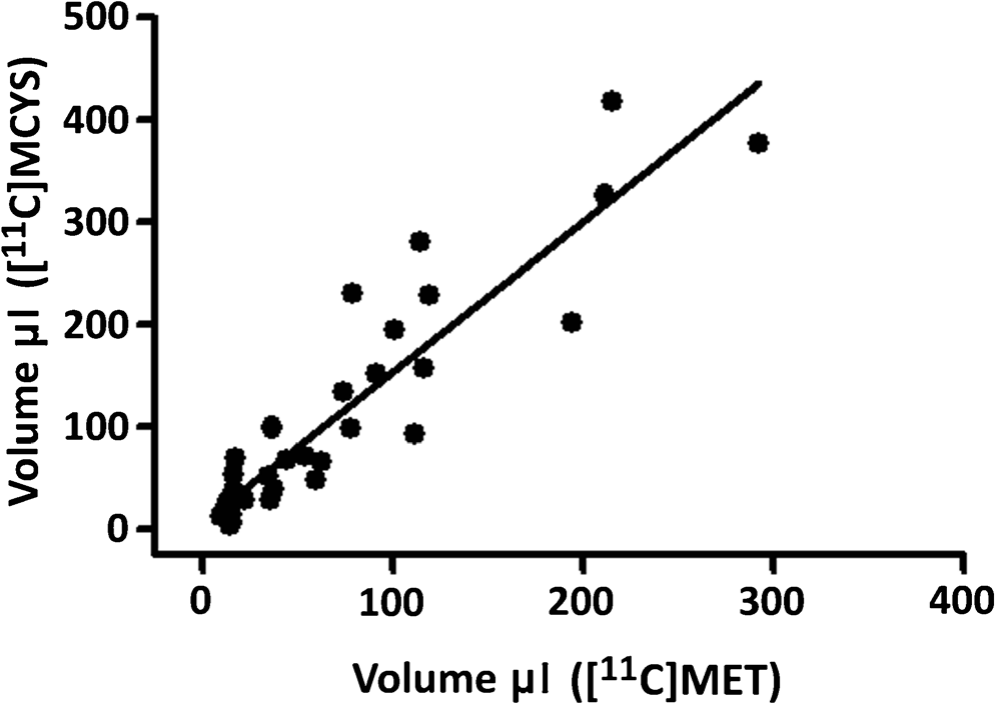
Tumor volume estimated from [^11^C]MCYS and [^11^C]MET scans (same animal, same tumor, same scanning day). The volume estimated with [^11^C]MCYS was on average 47 % larger.

Mean tumor sizes estimated from [^11^C]MCYS or [^11^C]MET scans were linearly related (*r* = 0.92, *P* < 0.0001. Fig. 3), but the size estimated in a [^11^C]MCYS scan was 47 % greater than in a [^11^C]MET scan (slope of the regression 1.47 ± 0.11).

### Group 3

At 1 day after irradiation, the tumor uptake of [^11^C]MET was already significantly reduced (SUVmean by 17 %, SUVmax by 30 %), in contrast to the uptake of [^11^C]MCYS (SUVmean − 4 %, SUVmax − 7 %, compare Tables 2 and 3). However, at 4 days after irradiation, the uptake of both tracers showed a similar decline (SUVmean 13 to 16 %, SUVmax 30 to 32 %). Radiotherapy had no significant impact on the uptake of [^11^C]MET or [^11^C]MCYS in the healthy brain (*P* = NS, both at 9 and 12 days).

**Table 3:** Tracer uptake in rats which received radiotherapy (on day 8)

Tracer uptake	Tumor					*N*	Brain	
Time	*N*	[^11^C]MET SUVmean	[^11^C]MCYS SUVmean	[^11^C]MET SUVmax	[^11^C]MCYS SUVmax		[^11^C]MET SUVmean	[^11^C]MCYS SUVmean
6 days p.i.	7	0.92 ± 0.10	1.02 ± 0.05	1.14 ± 0.20	1.18 ± 0.15	7	0.57 ± 0.03	0.81 ± 0.05
9 days p.i.	7	1.04 ± 0.12^@^	1.19 ± 0.03^&^	1.34 ± 0.28^@^	1.67 ± 0.14	7	0.63 ± 0.07	0.85 ± 0.04
12 days p.i.	7	1.03 ± 0.09^@^	1.14 ± 0.05*	1.24 ± 0.21^#^	1.44 ± 0.19*	7	0.64 ± 0.05	0.85 ± 0.04
Uptake ratios		Tumor-to-brain						
	*N*	[^11^C]MET Mean ratio	[^11^C]MCYS Mean ratio	[^11^C]MET Max ratio	[^11^C]MCYS Max ratio			
6 days p.i.	7	1.61 ± 0.15	1.27 ± 0.08	1.81 ± 0.31	1.37 ± 0.22			
9 days p.i.	7	1.66 ± 0.08*	1.41 ± 0.05	1.88 ± 0.18*	1.84 ± 0.15			
12 days p.i.	7	1.62 ± 0.19^#^	1.33 ± 0.04*	1.76 ± 0.24*	1.56 ± 0.12			

See the footer of Table 2 for used abbreviations

*P < 0.0001, ^#^ *P* < 0.001, ^@^ *P* < 0.005, and ^&^ *P* < 0.01 indicate significant differences between irradiated (Table 3) and untreated (Table 2) rats

**Fig. 4:**
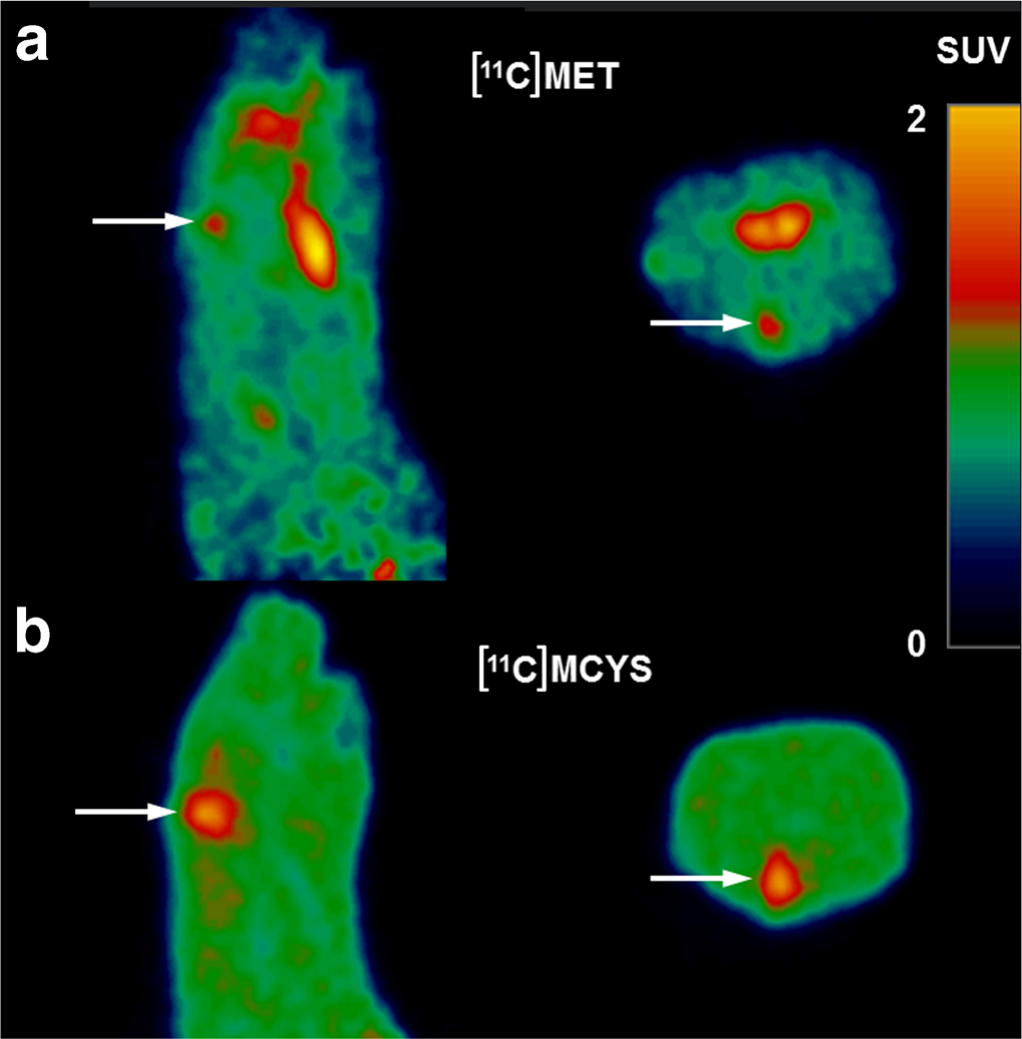
Sagittal and transaxial PET images of a single rat made 1 day after irradiation (9 days after inoculation of tumor cells). Arrows point to the position of the tumor. The estimated tumor volume (area with increased tracer uptake) is much larger after injection of (**b**) [^11^C]MCYS than after injection of (**a**) [^11^C]MET.

Tumor sizes estimated from [^11^C]MET and [^11^C]MCYS scans in untreated animals were closely correlated (Fig. 3), but [^11^C]MCYS indicated a larger tumor volume than [^11^C]MET in animals treated with radiotherapy (Table 4, Fig. 4). At 1 day after irradiation, [^11^C]MCYS showed a volume with increased uptake that was even larger than the tumor volume observed in untreated rats (Table 4). However, [^11^C]MCYS indicated a strong reduction in (apparent) tumor volume between 9 and 12 days (*i.e.*, between 1 and 4 days after irradiation), whereas [^11^C]MET did not show any significant change in tumor volume during this period. At 12 days p.i., a therapy-related suppression of tumor growth was detected with both tracers, whereas untreated gliomas continued to grow exponentially (Table 4). Tumor volume doubling times estimated from [^11^C]MET and [^11^C]MCYS scans were 2.3 and 2.1 days, respectively.

**Table 4:** Impact of radiotherapy (day 8) on estimated tumor volume (in μl)

Time	Number	Untreated [^11^C]MET	Irradiated [^11^C]MET	Untreated [^11^C]MCYS	Irradiated [^11^C]MCYS
6 days p.i.	7	17 ± 8	17 ± 6	28 ± 18	20 ± 11
9 days p.i.	7	68 ± 33	35 ± 13^@^	112 ± 90^!^	197 ± 86^$!^
12 days p.i.	7	147 ± 83	30 ± 14*	278 ± 147^!^	131 ± 103^&%^

*P.i.* post inoculation

*P < 0.0001, ^@^ *P =* 0.005, ^&^ *P* < 0.01 and ^$^ *P* < 0.05 indicate significant differences between untreated and irradiated rats

^!^ *P* < 0.005, ^%^ *P* < 0.01 indicate significant differences between [^11^C]MET and [^11^C]MCYS

### Groups 4 and 5

Both radiolabeled probes showed some accumulation in striatal lesions but similar uptake after injection of saline or LPS (Table 5). Their lesioned-to-healthy striatum ratios were not significantly different (Table 5). SUV values of [^11^C]MCYS, both in lesioned striata and in the healthy brain, were slightly higher than those of [^11^C]MET.

**Table 5:** Tracer uptake in animals with neuroinflammation

Tracer uptake	Lesioned striatum				Intact striatum			
	[^11^C]MET SUVmean	[^11^C]MCYS SUVmean	[^11^C]MET SUVmax	[^11^C]MCYS SUVmax	[^11^C]MET SUVmean	[^11^C]MCYS SUVmean	[^11^C]MET SUVmax	[^11^C]MCYS SUVmax
LPS-injected (n = 9)	0.73 ± 0.08	1.03 ± 0.07*	0.86 ± 0.12	1.22 ± 0.19^#^	0.62 ± 0.06	0.85 ± 0.06*	0.65 ± 0.06	0.89 ± 0.05*
Saline-injected (*n* = 9)	0.70 ± 0.06	0.97 ± 0.13^#^	0.86 ± 0.16	1.14 ± 0.18^@^	0.61 ± 0.05	0.84 ± 0.10*	0.65 ± 0.06	0.87 ± 0.10*
Uptake ratios	Lesioned-to-intact				Lesion volume (μl)			
	[^11^C]MET Mean ratio	[^11^C]MCYS Mean ratio	[^11^C]MET Max ratio	[^11^C]MCYS Max ratio	[^11^C]MET	[^11^C]MCYS		
LPS-injected (n = 9)	1.17 ± 0.07	1.21 ± 0.06	1.32 ± 0.18	1.37 ± 0.20	13 ± 5	44 ± 27^&^		
Saline-injected (n = 9)	1.15 ± 0.04	1.16 ± 0.06	1.33 ± 0.20	1.31 ± 0.20	13 ± 4	18 ± 10^!^		

*P ≤ 0.0001, ^#^ *P* < 0.0005, ^@^ *P* < 0.005, and ^&^ *P* < 0.01 indicate significant differences between [^11^C]MET and [^11^C]MCYS

^!^ *P* < 0.0001 indicates significant difference between LPS and saline

Although the lesioned-to-healthy striatum ratios of the tracers were not significantly different, the lesion volume estimated for LPS-injected striatum in [^11^C]MCYS scans was more than 3-fold higher (*P* < 0.01) than in [^11^C]MET scans (Table 5). [^11^C]MCYS scans indicated that injection of LPS causes damage to a larger brain area than injection of saline (*P* < 0.0001). This difference between saline and LPS was not visualized by [^11^C]MET (Table 3).

## Discussion

[^11^C]MCYS has been evaluated in a heterotopic mouse model of hepatocellular carcinoma and in a human PET study involving a patient with recurrent glioma [5]. Here, we compared the uptake of [^11^C]MCYS and [^11^C]MET in an orthotopic glioma model. The rat model which we employed mimics the invasion and growth, as well as the neuroinflammation and leukocyte infiltration which occur in a human glioblastoma [9, 13]. Our pilot study (group 1) indicated rapid growth of implanted gliomas. Thus, each rat in the following groups was scanned at rather short intervals (6, 9, and 12 days) after inoculation.

The PET studies indicated a similar uptake of the two tracers in untreated gliomas at 6, 8, and 9 days and a slightly higher uptake of [^11^C]MCYS at 12 days after inoculation (Table 2). However, [^11^C]MCYS showed also higher uptake in healthy brain tissue than [^11^C]MET (Table 2). For this reason, tumor-to-healthy brain ratios of [^11^C]MCYS were 19 to 26 % lower than of [^11^C]MET (Table 2). At short intervals between the inoculation of tumor cells and the PET scan (6 days), [^11^C]MET was strongly accumulated in the wound on top of the skull and also in salivary glands and nasal epithelium, in contrast to [^11^C]MCYS (Table 2, Fig. 1). The strong accumulation of [^11^C]MET in healing wounds and at the injection spot of the brain was a complicating factor in the detection and delineation of small tumors. Methionine is not only a substrate for protein synthesis but is also involved in transmethylation [14]. The label of [^11^C]MET may therefore end up in various phospholipids, DNA, and RNA [2], in contrast to the label of [^11^C]MCYS which is not incorporated into macromolecules during a PET scan [5]. This difference in the metabolism of the two tracers may explain why [^11^C]MET (but not [^11^C]MCYS) accumulates in healing wounds.

We also examined the impact of radiotherapy on tracer uptake in gliomas. Tumor-bearing rats were scanned 1 and 4 days after the application of a single X-ray fraction of 25 Gy on day 8 after inoculation. The uptake of both tracers showed a significant decline after radiotherapy (Table 3) which reflects a decrease of tumor metabolism. In untreated animals, [^11^C]MCYS indicated 47 % larger tumor volumes than [^11^C]MET (Fig. 3), but the close correlation between [^11^C]MCYS and [^11^C]MET volumes was lost after irradiation. In irradiated animals, [^11^C]MCYS indicated 5- to 6-fold larger tumor volumes than [^11^C]MET (Table 4). The tumor volume observed in [^11^C]MCYS scans 1 day after irradiation was even much (1.8-fold) larger than the tumor volume observed with the same tracer in untreated rats (Table 4). The acute increase in apparent tumor volume after irradiation that we observed in [^11^C]MCYS but not in [^11^C]MET scans seemed therefore to be related to a treatment-induced artifact (increased blood flow, increased leakiness of the blood-brain barrier, brain-infiltrating leukocytes) rather than the presence of viable tumor cells.

Since apparent tumor volumes after radiotherapy may be affected by tracer uptake in activated microglia, we examined the uptake of [^11^C]MET and [^11^C]MCYS in a neuroinflammation model. The LPS model which we employed is associated with activated microglia and infiltration of leukocytes after 3 days [10]. [^11^C]MCYS showed a higher uptake than [^11^C]MET both in LPS-injected and saline-injected striatum, but the uptake of [^11^C]MCYS in healthy brain tissue was also higher. The lesioned-to-intact striatum ratios of the two tracers were therefore identical (Table 4). Tracer uptake in LPS-injected and saline-injected striatum was similar. This observation suggests that both tracers show negligible accumulation in activated microglia. However, an interesting difference was noted when the *volume* with significant tracer accumulation was considered. [^11^C]MET and [^11^C]MCYS indicated identical lesion volumes in saline-injected animals, but [^11^C]MCYS indicated a larger volume for LPS-injected striatum than [^11^C]MET. The difference between LPS and saline was thus detectable in [^11^C]MCYS but not in [^11^C]MET scans (Table 4). Since the PET scans in the neuroinflammation model did not suggest a significant accumulation of [^11^C]MCYS in leukocytes or activated microglia, radiotherapy and LPS injection appear to cause secondary damage to the area surrounding the tumor or primary lesion which facilitates uptake of [^11^C]MCYS but not of [^11^C]MET. The mechanism underlying this facilitation is unknown and can only be clarified by further study, *e.g.*, by pathologic analysis of the area with increased [^11^C]MCYS uptake, 1 day after tumor irradiation.

## Conclusion

[^11^C]MCYS has been proposed as an oncologic PET tracer with greater tumor selectivity than [^11^C]MET. In support of this claim, we observed a strong accumulation of [^11^C]MET in salivary glands, Harderian glands, nasal epithelium, and healing wounds whereas [^11^C]MCYS showed little accumulation in such tissues. However, (1) the tumor-to-brain contrast in [^11^C]MCYS scans was 19 to 26 % lower than the tumor-to-brain contrast of [^11^C]MET. This difference in image contrast was due to a higher uptake of [^11^C]MCYS in brain tissue; (2) an acute, 2-fold increase of the apparent tumor volume was observed in [^11^C]MCYS scans after irradiation which appeared to be related to a treatment-induced artifact; (3) the decrease of tumor metabolism induced by radiotherapy was detected earlier in [^11^C]MET than in [^11^C]MCYS scans; and (4) both [^11^C]MET and [^11^C]MCYS showed negligible accumulation in LPS-injected striatum, *i.e.*, in activated microglia. For these reasons, [^11^C]MCYS and [^11^C]MET appear to reflect different aspects of *in vivo* biology. [^11^C]MCYS may offer the advantage of lower background accumulation in extracranial tissue whereas [^11^C]MET may exhibit a lower normophysiological signal in the brain. Since the current data were acquired in anesthetized animals, tracer distribution may be different in the absence of anesthesia. Future studies should be aimed at elucidating the processes affecting the uptake of both amino acids and identifying the advantage of one compound over another in various circumstances.

## Electronic supplementary material

ESM 1 (PDF 101 kb)
