# Supplementary material for: PET Imaging with S-[11C]Methyl-L-Cysteine and L-[Methyl-11C]Methionine in Rat Models of Glioma, Glioma Radiotherapy, and Neuroinflammation
Source: Mol Imaging Biol. 2017 Oct 30;20(3):465–72. doi: 10.1007/s11307-017-1137-z (PMC5938303; doi:10.1007/s11307-017-1137-z)
Supplement: Supplementary file 1 — (PDF 101 kb) [file 11307_2017_1137_MOESM1_ESM.pdf]

Electronic Supplementary Material

PET Imaging with *S*-[^{11}C]Methyl-L-Cysteine and L-[*Methyl*-^{11}C]Methionine in Rat Models of Glioma, Glioma Radiotherapy and Neuroinflammation

Journal: Molecular Imaging and Biology

Andrea Parente[a], Aren van Waarde[a], Alexandre Shoji[a,b], Daniele de Paula Faria[a,b], Bram Maas[a], Rolf Zijlma[a] , Rudi A.J.O. Dierckx[a], Johannes A. Langendijk[c], Erik F.J. de Vries[a] and Janine Doorduin[a]

[a] University of Groningen, University Medical Center Groningen, Nuclear Medicine and Molecular Imaging, Hanzeplein 1, 9713 GZ Groningen, Netherlands

[b] University of Sao Paulo, Faculdade de Medicina, Hospital das Clinicas, Laboratory of Nuclear Medicine (LIM43), Sao Paulo, SP, Brazil

[c] University of Groningen, University Medical Center Groningen, Department of Radiation Oncology, Hanzeplein 1, 9713 GZ Groningen, Netherlands.

Corresponding author: A. van Waarde, Ph.D., University of Groningen, University Medical Center Groningen, Nuclear Medicine and Molecular Imaging, Hanzeplein 1, 9713 GZ Groningen, The Netherlands. Telephone: +31-50-3613215, Telefax: +31-50-3611687, E-mail: a.van.waarde@umcg.nl

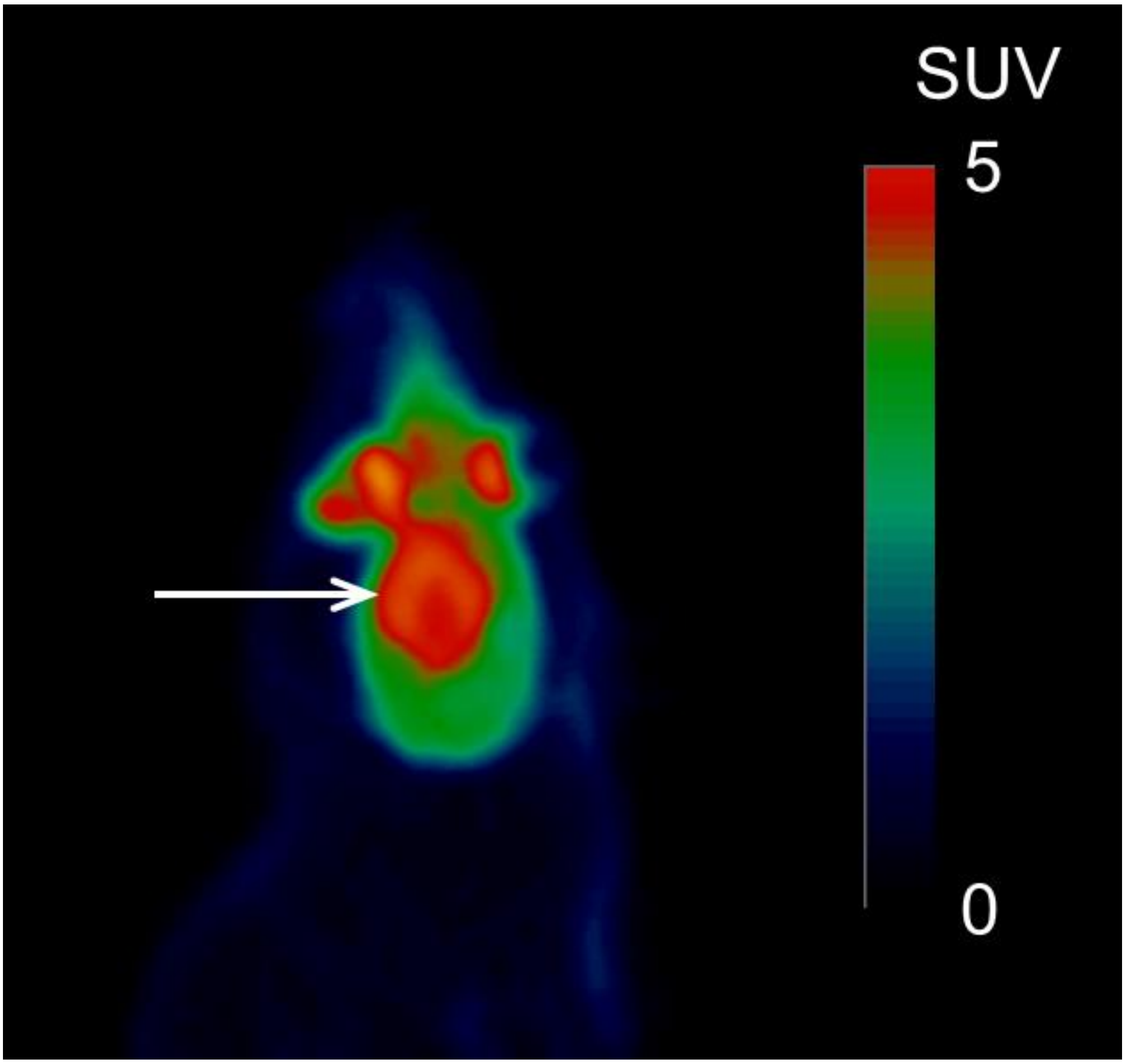

Figure S1: Coronal [^{18}F]FDG PET image of the brain of a rat, made 15 days after inoculation of C6 cells in the right striatum. The position of the tumor is indicated by the arrow. At this interval, the tumor had acquired a very large size, fills almost an entire cerebral hemisphere and begins to invade the rest of the brain.
